# Effects of Memantine in a Mouse Model of Postoperative Cognitive Dysfunction

**DOI:** 10.3390/bs9030024

**Published:** 2019-03-06

**Authors:** Ahmad Almahozi, Mohamed Radhi, Suja Alzayer, Amer Kamal

**Affiliations:** Physiology Department, College of Medicine and Medical Sciences, Arabian Gulf University, P.O. Box 26671, Manama 1111, Bahrain; suja.alzayer@gmail.com (S.A.); amerha@agu.edu.bh (A.K.)

**Keywords:** postoperative cognitive dysfunction, POCD, memantine, Alzehimer’s, neuroinflammation, mice

## Abstract

Persistent impairment in cognitive functioning postoperatively is reported by clinical and animal studies, and is labeled as postoperative cognitive dysfunction (POCD). Evidence points to an exaggerated neuroinflammatory response resulting from peripheral systemic inflammation after surgery, with subsequent cytokine-induced glutamatergic excitotoxicity and synaptic impairment. These immunological changes, among many others, are also observed in Alzheimer’s disease. Memantine is an N-methyl-D-aspartate receptor (NMDAR) antagonist commonly used to treat Alzheimer’s disease. Surprisingly, little research exists on the role of memantine in preventing POCD. The purpose of this study is to investigate the effects of memantine on a spectrum of cognitive functions postoperatively. Mice were divided into 3 groups and each received treatment for 4 weeks. Placebo groups received a placebo then underwent either a sham procedure or a laparotomy procedure. The memantine group received memantine hydrochloride then underwent a laparotomy procedure. Cognitive tests were performed on postoperative days (POD) 1 and 7. Compared to sham-operated mice, placebo groups that underwent a laparotomy procedure showed impaired memory in the Morris water maze test, higher anxiety-like behavior in the open field and the elevated plus maze tests, increased depression-like behavior in the tail suspension test, and lack of preference for social novelty in the three-chamber test. On the other hand, memantine-treated mice that underwent a laparotomy procedure showed enhanced memory on POD7, improved depression-like behavior on POD1 and POD7, enhanced preference for social novelty on POD1, and no improvement in anxiety-like behavior. These findings suggest a potential protective effect of memantine in mice postoperatively on memory, depression-like behavior, and preference for social novelty.

## 1. Introduction

The allegations and remarks raised by family and friends of patients that underwent surgery, such as “He’s never been the same since his operation” or “She’s lost all interest in the family since …”, have led Bedford [1] to publish the first comprehensive report that describes cognitive decline after surgery in the elderly. Bedford has attributed the phenomenon mostly to anesthesia, and hypoxic intervals during anesthesia and surgery. Majority of studies since then [2,3,4,5,6,7] have focused on cardiac surgeries, especially cardiopulmonary bypass surgery, presumably due to the potential for hypoxic/hypoperfusion injury and microemboli-related ischemia during these types of surgeries. However, a large study published in 1998 [8] established long-term cognitive dysfunction (detected at 3 months after surgery) in patients undergoing non-cardiac surgery (major abdominal and orthopedic surgeries), and concluded that the occurrence of this dysfunction is not related to hypotension or hypoxemia, but to anesthesia and surgery, with advanced age being the strongest risk factor. Subsequent studies have also determined that this complication, now termed postoperative cognitive dysfunction (POCD), could occur after minor surgery [9], and is not related to the depth of anesthesia [10,11]. Moreover, POCD has been shown to increase the risk of developing dementia 3–5 years after surgery [12], and increase the risk of death in the first year following surgery [13].

To distinguish it from postoperative delirium, which occurs in the first few days following surgery, POCD is defined as persistent cognitive impairments (lasting for more than one week) after surgery [14]. Also, the acute and transient cognitive disturbances in delirium may involve fluctuating mental status, while POCD alterations are more subtle, and involve memory, concentration, language comprehension, and a decreased ability to process information, without changes in the levels of consciousness [15,16,17,18].

The pathophysiology underlying POCD is not yet fully understood. Accumulating evidence point to the role of peripheral systemic inflammation (due to surgical trauma) in causing exaggerated inflammatory responses in the brain [14], as shown by patient studies [19,20,21] and animal studies, particularly in the hippocampus [22,23,24]. Many of the immunological changes observed in POCD, including tau phosphorylation, cytokine-induced glutamatergic excitotoxicity, and most importantly, beta-amyloid accumulation, are similar to the changes found in Alzheimer’s disease [22,25,26]. Therefore, both entities could be viewed as a disease continuum [15].

Currently, there is no adequate treatment or prophylaxis for POCD [14,15], and management usually revolves around identifying risks, and non-pharmacological preventive measures [27,28,29]. Memantine is an N-methyl-D-aspartate receptor (NMDAR) antagonist that is shown to improve cognitive function in transgenic animal models of Alzheimer’s disease [30,31,32], and its use is widely supported by human clinical trials in treating moderate to severe Alzheimer’s disease [33,34]. Surprisingly, little research exists on the role of memantine in the management of POCD. The purpose of this study is to investigate the effects of memantine on a spectrum of cognitive functions in a mouse model of POCD.

## 2. Materials and Methods

### 2.1. Animals

Male BALB/C mice (n = 60), aged 4–6 months were used. The animals weighed 25–36 g at the beginning of the experiment. All animals were housed individually in cages of 52 cm in length × 30 cm in width × 21 cm in height, with soft bedding. The animal colony was maintained at 22 ± 2 °C and a relative humidity of 40–60%, on a 12-hour light/dark cycle (lights on at 6:00 A.M). All mice were allowed ad libitum access to laboratory chow and tap water, and were given at least 1 week to acclimate to colony conditions before experimentation began.

**Figure 1:**
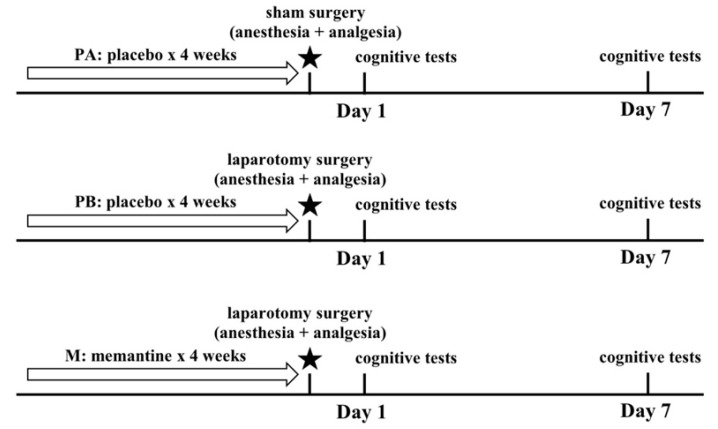
Experimental design. Placebo A (PA) group received placebo for 4 weeks, then underwent a sham procedure. Placebo B (PB) group received placebo for 4 weeks, then underwent a laparotomy procedure. Memantine (M) group received memantine hydrochloride for 4 weeks, then underwent a laparotomy procedure. From each group, half of the animals underwent cognitive testing on postoperative day 1 (POD1), and the other half underwent cognitive testing on postoperative day 7 (POD7).

The animals were randomly divided into three groups (Figure 1). Placebo-A (PA) group (n = 20, from 10 different mothers) received placebo for 4 weeks, then underwent a sham procedure. Placebo-B (PB) group (n = 20, from 10 different mothers) received placebo for 4 weeks, then underwent a laparotomy procedure. Memantine (M) group (n = 20, from 10 different mothers) received memantine hydrochloride for 4 weeks, then underwent a laparotomy procedure. For each group, half of the animals (n = 10) underwent behavioral testing on postoperative day 1 (POD-1), and the other half (n = 10) underwent behavioral testing on postoperative day 7 (POD-7). The weights (g) of the animals were measured during the treatment period at days 7, 14, 21, and 28, and after the operations on days 1, 3, and 7.

All experimental procedures and behavioral tests in the project (55-PI-02/16) were approved by the Research Ethics Committee, the Arabian Gulf University, Manama, Bahrain. All efforts were made to minimize the number of animals used, and the animals were handled in accordance with good animal practice.

### 2.2. Treatment

For the memantine group (M), memantine hydrochloride (Lundbeck A/S, Denmark) was administered daily for 4 weeks, as a solution in physiological saline via oral gavage, at a therapeutic dose of 30 mg/kg/day. A steady-state plasma drug level of memantine of around 1 µmol/L in mice is considered therapeutic [35,36,37], and a dose of 30 mg/kg/day is shown to be sufficient to reach that level [30]. To control for the effects of stress, placebo groups (PA and PB) received physiological saline (0.9% sodium chloride, Argyle, Covidien, USA) via oral gavage for 4 weeks.

### 2.3. Surgical Procedure

Laparotomies and sham surgeries were performed using aseptic procedures under general anesthesia with intraperitoneal injections of ketamine (Hikma Pharmaceuticals PLC, UK) at a dose of 1.0 mg/10 g body weight and xylazine (Bomac Laboratroies Ltd., New Zealand) at a dose of 0.1 mg/10 g body weight, following a previously described method [38].

After the induction of anesthesia, the abdominal region was shaved and thoroughly cleaned with 70% ethanol (Green Cross, Philippines) and surgical scrub (Povidone-iodine 10%, Purdue Products L.P., Stamford, CT). Approximately 0.5 cm below the left rib in the left upper abdominal quadrant, a 1.5 cm vertical incision was made, penetrating the skin and the peritoneal cavity. A sterile probe was inserted into the opening and manipulated the viscera and musculature for 1 min. Sterile dissolvable sutures (Vicryl 3-0, 70 cm; Ethicon) were used to suture the abdominal muscles. The skin was closed with silk thread sutures. To reduce the risk of infection, the wound was dressed with Polysporin (Pfizer). Sham-operated mice (PA group) were anesthetized, and the abdominal area was shaved and cleaned as described above. They remained on ketamine and xylazine for the same amount of time as their surgical counterpart (about 10 min). Upon recovery from anesthesia, all animals received buprenorphine (Napp Pharmaceuticals Ltd., Cambridge, UK) at a dose of 1.0 mg/10 g body weight subcutaneously for analgesia. All procedures were performed by one person to limit variability. In the postoperative period, surgical wounds were inspected daily for wound dehiscence or discharge.

### 2.4. The Tests

Behavioral tests were conducted between 6:00 A.M and 6:00 P.M. All sessions were videotaped, and were conducted and scored blindly. To eliminate possible inter-observer variability, each parameter was scored by one observer for all mice.

#### 2.4.1. The Morris Water Maze

Each animal underwent 5 training trials per day for the first day in the Morris water maze, followed by 3 trials in the second day, and a final probe trial on the third day, as previously described [39,40].

The maze consisted of a large circular swimming pool, measuring 140 cm in diameter and 50 cm in height, filled to a depth of 30 cm, and the water was maintained warm at 24 °C. The pool was placed in a darkened room that is illuminated by sparse red light, with surrounding extra-maze visual cues. Two imaginary diagonal lines divided the pool into 4 equal quadrants. Mice were given acquisition trials to learn the position of a hidden platform (diameter 12 cm), submerged 2 cm below the water surface, in the center of one quadrant. Performance in the trials was averaged to yield one single data point per mice per day. On each trial, the mice were released into the water facing the wall of the pool from one of four randomly assigned positions on the perimeter of the pool (N, W, S, E), and were allowed a maximum of 120 seconds per session to find the hidden platform. They were allowed to remain on the platform for 30 seconds in between sessions. If an animal failed to find the platform within the allowed time, an experimenter would gently guide the animal to the platform.

The position and movement of the animals in the pool were recorded and analyzed using the ANY-maze video-tracking system (Stoelting Co, Wood Dale, IL, USA). Outcome measures were time spent to reach the platform (latency time), distance swum to reach the platform, and speed of swimming. On the third day, a final probe trial was conducted in which the platform was removed and each animal was allowed to swim for 60 seconds. The time spent in the zone that previously contained the platform was analyzed, and the selective search strategy was indicated if animals performed significantly above chance (25%).

#### 2.4.2. The Open Field Test

Locomotor function and anxiety were assessed using the open field test, as described previously [41]. A square wooden open field (44 × 44 × 32 cm) was subdivided into 16 even squares with thin white stripes. Each mouse was placed in next to the wall of the arena, facing away from the experimenter. Behavior was recorded for 10 min.

Outcome measures were distance traveled (indicated by number of line crossings), frequency of central zone entry, and time spent in the central zone (the central four squares). Distance traveled reflected the general motor function. Time spent in the central zone and frequency of central area entry were measures of anxiety. At the end of each trial, the arena was cleaned using 70% ethanol to prevent olfactory cue bias.

#### 2.4.3. The Elevated Plus-Maze Test

Anxiety-like behavior was assessed using the elevated plus-maze test, as described previously [42]. The apparatus comprised of four arms; two open, unprotected arms (25 × 5 cm) surrounded by small walls (3 mm high) to prevent the animals from falling, and two enclosed, protected arms (25 × 5 cm) surrounded by high walls (15 cm high). All arms were elevated 55 cm above the floor. Arms of the same type were located opposite to each other, with an empty square (5 × 5 cm) in the center of the maze. The test started by individually placing each mouse in the central square facing one of the closed arms, and allowing it to freely explore the apparatus for 10 min.

Measures recorded were the number of entries into an arm, and the time spent in open and closed arms. Percentage of entries into open arms, time spent in open arms, and total number of entries were analyzed. Entry into an arm was defined as all four limbs in an arm. Anxiety-like behavior was indicated by decreased number of entries into the open arms, and spending less time in the open arms. Total number of entries into open and closed arms was a measure of general activity. After each session, the apparatus was cleaned thoroughly with 70% ethanol.

#### 2.4.4. The Tail-Suspension Test

The tail-suspension test was used to assess depression-related behavior, as described previously [43]. A rectangular box (walls made of Plexiglas, measuring 45 × 24 × 24 cm) was used. On top of the box, a suspension bar (made of aluminum, measuring 1 × 1 × 55 cm) was placed and used to suspend the tail of the mouse. Each mouse was suspended separately in the middle of the box, using an adhesive black tape applied to the end of the tail (with 2–3 millimeters remaining outside of the tape), and the free end of the tape was attached to the middle of the suspension bar. The box was sufficiently sized so the animal could not make contact with the walls. The approximate distance between the mouse’s nose and the apparatus floor was 10–15 cm. Every session lasted for 6 min, and was recorded using a video camera placed on a tripod in front of the box. At the end of every session, the adhesive tape was gently removed from the tail, and the suspension box was thoroughly wiped with 70% ethanol.

The time that each mouse spent as immobile was measured. Mobility included escape-related behaviors, such as trying to reach the walls of the apparatus and the suspension bar, strong shaking of the body, and movement of the limbs akin to running. Small movements that were confined to the front legs but without the involvement of the hind legs were not counted as mobility. Swinging movements resulting from previous bouts of mobility also were not counted as mobility. Lack of escape-related behavior is considered immobility, which is indicative of depression-like behavior.

#### 2.4.5. The Three-Chamber Test

The three-chamber test [44] was used to assess sociability and preference for social novelty. The apparatus consisted of a rectangular box (walls made of Plexiglas) with removable partitions separating the box into three chambers. Each chamber was 20 × 40 × 22 cm. The dividing walls have small square openings (5 cm x 3 cm) which allow the mouse to move freely from one chamber to another. Each side chamber contained a circular metal wire cage, large enough to hold a single mouse (11 cm high, 9 cm diameter, and bars spaced 0.5 cm apart). The test consisted of two sessions: session 1 (testing sociability), and session 2 (testing preference for social novelty). Animals used were subject mice (PA, PB, and M groups), and naïve “stranger” mice (of the same age, gender, and background as the subject mice) that had no prior contact with the subject mice. To allow adaptation, the subject mouse was placed in the center of the middle chamber and left to habituate for 5 min.

In session 1, an unfamiliar mouse (stranger 1) was placed inside the wire cage that was located in one of the side chambers. The placement of stranger 1 in the left or right side of the chamber was systematically altered between trials. The partitioning walls between the chambers were removed to allow free access for the subject mouse to explore each of the three chambers. Session 1 was recorded and continued for 10 min. The parameters assessed were time spent in each chamber and the total number of chamber entries.

After the end of session 1, session 2 started immediately with the placement of a second unfamiliar mouse (stranger 2) inside the other wire cage that was placed in the center of the opposite side chamber (which was previously empty during session 1). The subject mouse had the freedom to access all three chambers. The duration for session 2 was 10 min, and the same parameters were assessed as in session 1. After each session, all chambers were cleaned with 70% ethanol to prevent olfactory cue bias.

Normal sociability/social motivation (tested in session 1) was indicated by spending more time in the chamber containing stranger 1 compared to the chamber with empty cage. Intact social memory and predilection for novel experiences (tested in session 2) were indicated by spending more time in the chamber containing the newly encountered mouse (stranger 2) compared to the chamber containing the first, already-investigated mouse (stranger 1).

### 2.5. Data Analysis

Statistical analyses were performed using SPSS package (version PASW Statistic 18.0.3) and Microsoft Excel (version 15.41). All data are represented as mean ± standard error of the mean (SEM). Differences in the performance between and within groups were assessed using a one-way analysis of variance (ANOVA), followed by paired or unpaired post-hoc t-tests. Statistical significance was set at a *p* value of less than 0.05.

## 3. Results

### 3.1. Significant Weight Loss in Groups that Underwent Laparotomy Procedure Compared to Sham-Operated Mice

**Figure 2:**
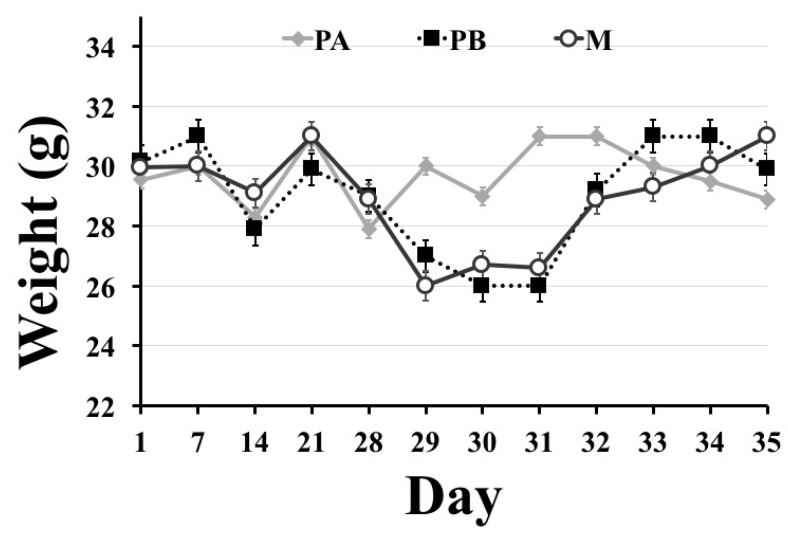
Changes in body weight (g). On postoperative days 1 and 3 (days 29 and 31 of the experiment), mice that underwent surgery (PB and M) had lower weights compared to sham-operated mice (PA). On postoperative day 7 (day 35 of the experiment), all groups had similar weights. Data shown as mean ± SEM.

Weight changes were recorded for all animals (Table 1 and Figure 2). No significant differences were found between the groups during the treatment period (days 1–28). On postoperative days 1 and 3 (days 29 and 31 of the experiment, respectively), the PA group had higher weights compared to PB and M groups. On postoperative day 7, the final day of the experiment (day 35), there were no significant differences between the groups. Additionally, surgical wounds were inspected daily in the postoperative period. No wound dehiscence or discharge were found in any of the animals.

### 3.2. Impaired Memory Following Surgery, with Improved Performance in Memantine Group on Postoperative Day 7

**Figure 3:**
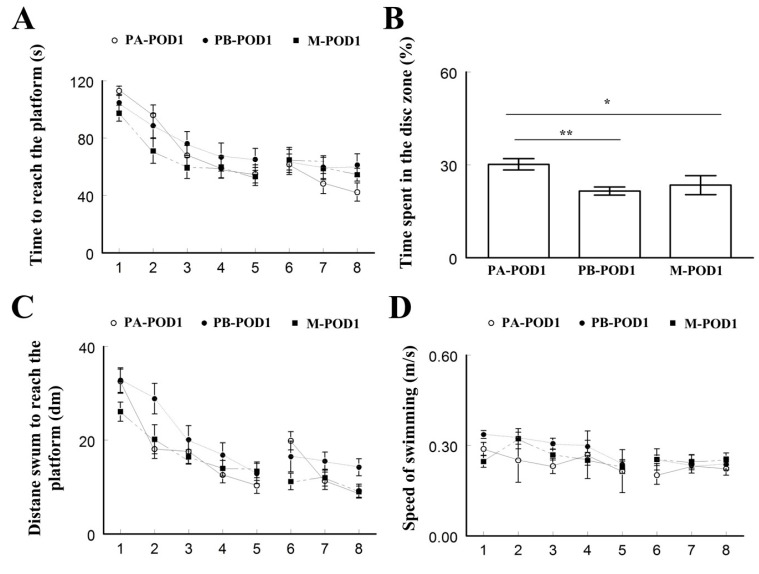
Morris water maze was used to assess for memory (postoperative day 1). (**a**) Time to reach the platform (latency); (**b**) time spent in the disc zone; (**c**) distance swum to reach the platform; and (**d**) speed of swimming. Sham-operated mice (PA-POD1) spent less time to reach the platform, and more time in the disc zone compared to mice that underwent surgery (PB-POD1 and M-POD1). PB-POD1 travelled more distance to reach the platform compared to the other two groups, with no significant differences in the speed of swimming between the three groups. Data shown as mean ± SEM. * *p* < 0.05, ** *p* < 0.01.

Memory and learning were assessed using the Morris water maze. On postoperative day 1 (Table 2), sham-operated mice (PA-POD1) spent less time to reach the platform (Figure 3A), and more time in the disc zone (Figure 3B) compared to mice that underwent surgery (PB-POD1 and M-POD1). PB-POD1 travelled more distance to reach the platform compared to the other two groups (Figure 3C), with no significant differences in the speed of swimming (Figure 3D).

**Figure 4:**
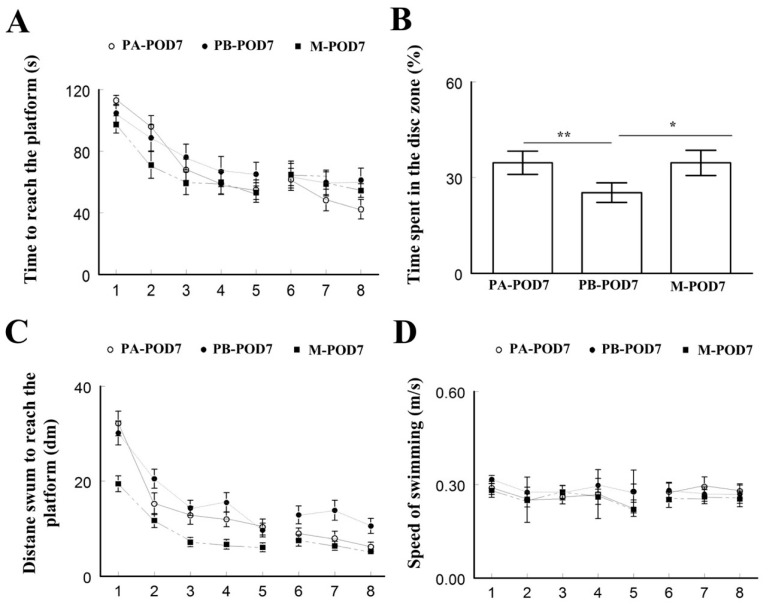
Morris water maze was used to assess for memory (postoperative day 7). (**a**) Time to reach the platform (latency); (**b**) time spent in the disc zone; (**c**) distance swum to reach the platform; and (**d**) speed of swimming. Sham-operated mice (PA-POD7) and memantine-treated mice (M-POD7) spent less time to reach the platform, more time in the disc zone, and travelled less distance compared to operated-mice that received placebo (PB-POD7). No significant differences were found in the speed of swimming between the three groups. Data shown as mean ± SEM. * *p* < 0.05, ** *p* < 0.01.

On postoperative day 7 (Table 2), sham-operated mice (PA-POD7) and memantine-treated mice (M-POD7) spent less time to reach the platform (Figure 4A), more time in the disc zone (Figure 4B), and travelled less distance (Figure 4C) compared to operated-mice that received placebo (PB-POD1). No significant differences were found in the speed of swimming (Figure 4D).

### 3.3. Presence of Anxiety-Like Behavior Following Surgery, with No Improvement in Memantine Groups

**Figure 5:**
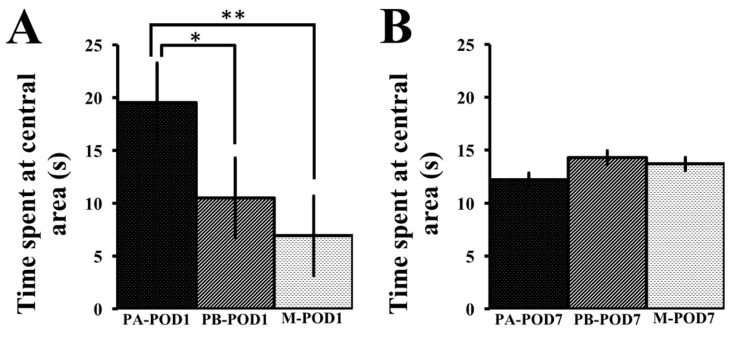
The open field test was used to assess for locomotor function and anxiety. (**a**) Time spent at central area in seconds (POD1); (**b**) time spent at central area (POD7). On postoperative day 1, sham-operated mice (PA-POD1) spent more time in the central area compared to operated-mice (PB-POD1 and M-POD1). On postoperative day 7, no significant differences were found between the three groups. Data shown as mean ± SEM. * *p* < 0.05, ** *p* < 0.01.

Locomotion and anxiety-like behavior were assessed using the open field test and the elevated plus-maze. In the open field test on postoperative day 1 (Table 3), no significant differences were found between PA-POD1, PB-POD1, and M-POD1 groups in the total distance traveled (number of lines crossed) or in the frequency of central area entry. However, PA-POD1 group spent significantly longer times in the central zone compared to the other groups (Figure 5A). On postoperative day 7 (Table 3), no significant differences were found in the total distance traveled, the frequency of central zone entry, and the total time spent in the central zone (Figure 5B).

**Figure 6:**
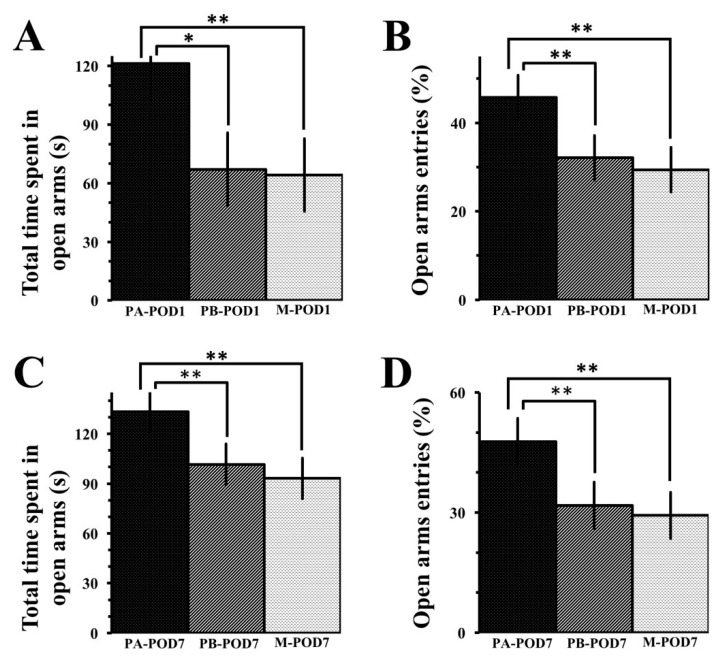
The elevated-plus maze was used to assess for anxiety-like behavior. (**a**) Total time spent in open arms (POD1); (**b**) open arms entries (POD1); (**c**) total time spent in open arms (POD7); (**d**) open arms entries (POD7). On postoperative days 1 and 7, sham-operated mice (PA-POD1 and PA-POD7) spent more time in the open arms and had higher percentages of open arm entries compared to operated-mice (PB-POD1, PB-POD7, M-POD1 and M-POD7. Data shown as mean ± SEM. * *p* < 0.05, ** *p* < 0.01.

In the elevated plus-maze on postoperative day 1 (Table 4), mice in the PA-POD1 group spent longer time in the open arms (Figure 6A), and had higher percentages of open arms entries (Figure 6B) compared to PB-POD1 and M-POD1 groups. No significant differences were found in the total number of arm entries. On postoperative day 7 (Table 4), PA-POD7 group spent longer time in the open arms (Figure 6C), and had higher percentages of open arms entries (Figure 6D) compared to the other two groups. No significant differences were found in the total number of arm entries.

### 3.4. Presence of Depression-Like Behavior Following Surgery, with Improvement in Memantine Groups

**Figure 7:**
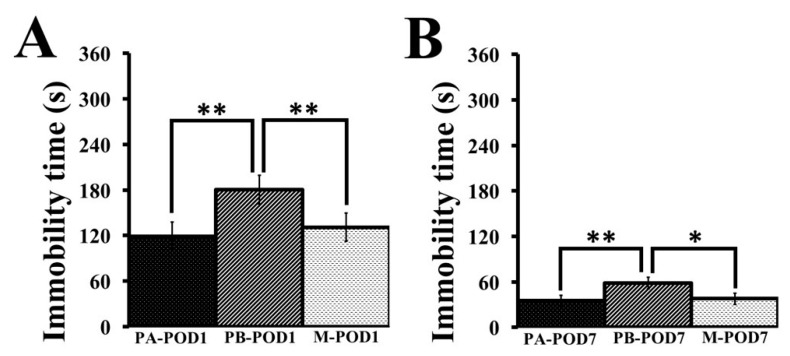
The tail-suspension test was used to assess depression-like behavior (postoperative days 1 and 7). (**A**) Immobility time (POD1); and (**B**) immobility time (POD7). On postoperative days 1 and 7, placebo groups (PB-POD1 and PB-POD7) showed depression-like behavior compared to sham-operated mice (PA-POD1 and PA-POD7) and memantine-treated mice (M-POD1 and M-POD7), as they had higher immobility times. Data shown as mean ± SEM. * *p* < 0.05, ** *p* < 0.01.

Depression-like behavior was assessed using the tail-suspension test. On postoperative days 1 (Figure 7A) and 7 (Figure 7B), placebo groups that underwent a laparotomy (PB-POD1 and PB-POD7) showed depression-like behavior compared to sham-operated mice (PA-POD1 and PA-POD7) and memantine-treated mice (M-POD1 and M-POD7), as they had higher immobility times (Table 5).

### 3.5. Improved Preference for Social Novelty with Memantine on Postopeartive Day 1

**Figure 8:**
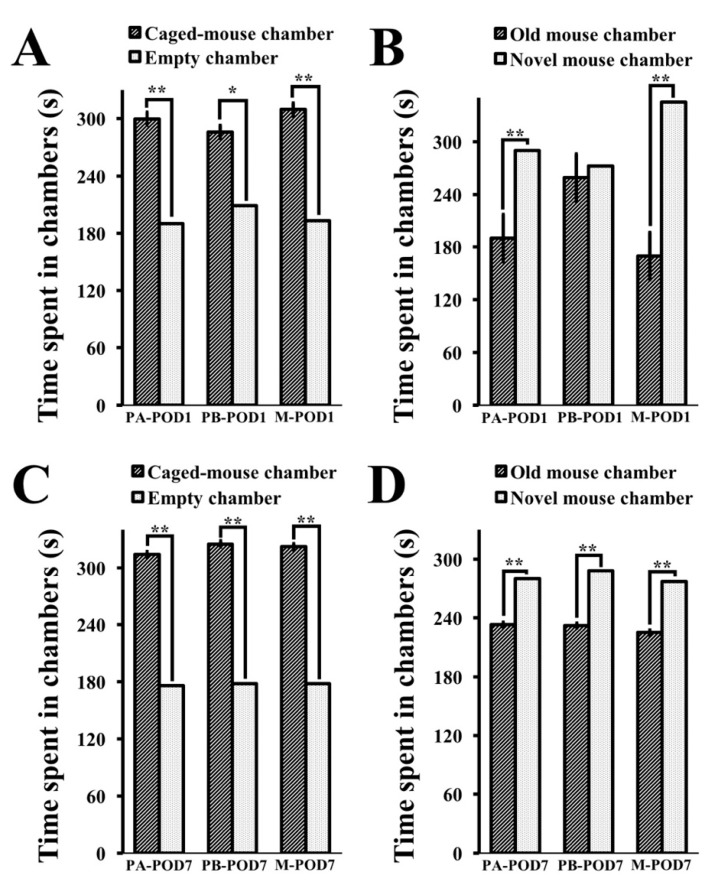
The three-chamber test was used to assess sociability and preference for social novelty. (**a**) Time spent in chambers in session 1 (POD1); (**b**) time spent chambers in session 2 (POD1); (**c**) time spent in chambers in session 1 (POD7); and (**d**) time spent in chambers in session 2 (POD7). On postoperative day 1, mice in all three groups showed normal sociability as they spent more time in the chamber containing the caged-mouse (session 1). Preference for social novelty (session 2) was observed in PA-POD1 and M-POD1 as mice spent more time in the chamber containing the new mouse, while PB-POD1 did show a preference for social novelty. On postoperative day 7, mice in all three groups showed normal sociability (session 1) and preference for social novelty (session 2). Data shown as mean ± SEM. * *p* < 0.05, ** *p* < 0.01.

In the three-chamber social apparatus, normal levels of sociability were observed on postoperative day 1 (Table 6), as mice in session 1 (Figure 8A) spent more time in the chamber containing the caged mouse than the empty one, in all three groups; PA-POD1, PB-POD1 and M-POD1. In session 2 (Figure 8B), PA-POD1 and M-POD1 demonstrated preference for social novelty, as mice spent more time in the chamber containing the novel mouse than the chamber containing the already investigated mouse. By contrast, mice in the PB-POD1 did not show preference for social novelty, as they did not spend significantly different times in the chambers in session 2. No significant differences were found in the number of entries to the two chambers for all groups, both in session 1 and 2 (Table 7).

On postoperative day 7 (Table 6), normal levels of sociability were observed in all three groups in session 1 (Figure 8C); and in session 2 (Figure 8D), with no significant differences in the number of entries to the chambers (Table 7).

## 4. Discussion

The purpose of this study is to examine the effect of memantine on a spectrum of cognitive functions postoperatively. Hovens and colleagues [14] point to a translational gap in postoperative cognitive dysfunction (POCD) research. POCD is often reported by clinical studies without specifying what cognitive functions or brain structures are involved. Memory, concentration, language comprehension, and information processing are commonly implicated, but only few studies employ neurophysiological testing to make distinctions between these cognitive domains (see [45,46,47] for examples). On the other hand, pre-clinical research using animal models mostly focused on memory and hippocampal injury, and neglected other aspects of cognition. Taking that into consideration, we attempted to investigate the effect of surgery on different aspects of cognition in mice. These include learning and memory, anxiety, locomotor function, depression, sociability and preference for social novelty.

With regards to body weight, mice that underwent surgery (PB and M groups) suffered from weight loss on postoperative days 1 and 3, but regained their weight on postoperative day 7. Weight loss in the first few days after surgery in POCD models is commonly reported [48,49], and it could be attributed to dehydration and fluid loss during surgery.

Learning and memory were assessed using the Morris water maze test. On postoperative days 1 and 7, mice that underwent surgery (PB-POD1 and PB-POD7) spent more time and travelled more distance to reach the platform, and spent less time in the disc zone in the probe test, compared to the groups that received anesthesia and analgesia without surgery (PA-POD1 and PA-POD7). Motor function was not affected, as all groups had similar speed of swimming. These findings suggest impaired learning and memory in mice following surgery. Several studies reported similar findings [48,50]. The duration of memory impairment following surgery varies in animal models of POCD. Most studies reported effects lasting between 1 and 7 days [23,24,38,51]. However, effects lasting for more than 1 week have also been reported [24,48,52]. In our study, memory was not tested beyond one week and, therefore, it is not possible, based on these findings, to determine whether these deficits are persistent or not.

The abdominal surgery performed on mice in this study is considered minor surgery. The surgical model used [38] was shown to lead to increased inflammatory changes in the hippocampus of aged mice (23–25 months old), but not in young adult mice (4–6 months old). Moreover, it did not result in significantly impaired performance in a reversal learning version of the Morris water maze. In our study, adult mice (aged 4–6 months) were tested. Interestingly, significant memory impairment was observed. This discrepancy could be attributed to the version of the Morris water maze test used. The standard Morris water maze, which tests spatial learning through visual cues with a probe test, is considered reflective of hippocampal function [53,54]. On the other hand, some variations of the Morris water maze, such as reversal tasks, are thought to be independent of hippocampal function [54,55]. Our findings support previous research, which have suggested that hippocampal-dependent memory is specifically susceptible to surgery-induced impairments [23,48,51,52,56].

Anxiety may inhibit exploratory behavior in mice [57], as well as performance in other cognitive tests. Anxiety-like behavior was assessed using the open field test and the elevated-plus maze. On postoperative day 1, sham-operated mice (PA-POD1) spent more time in the central area in the open field, and more time in the open arms of the elevated-plus maze with a higher number of entries to the open arms, compared to mice that underwent surgery (PB-POD1). On postoperative day 7, no significant differences were found in the open field test. However, sham-operated mice (PA-POD7) spent more time in the open arms of the elevated-plus maze with higher number of entries to the open arms compared to their surgical counterpart (PB-POD7). Collectively, these observations may reflect anxiety-like behavior and reduced interest in the environment. Several studies reported anxiety-like behavior following surgery [48,58,59,60], but mostly in the first two postoperative days. Because of that, anxiety in this context is often viewed as a transient response to acute illness or trauma [48,61,62], rather than a persistent cognitive deficit related to POCD. In our experiment, however, anxiety was observed on days 1 and 7 postoperatively. It is unclear whether this anxiety reflects a sickness-response to trauma, or is related to inflammatory changes in the brain, as no inflammatory assays were performed.

Decreased social activity following surgery has been reported previously by human studies [58,63], and animal models of peripheral inflammation [64,65] However, there is a lack of studies concerning sociability and social novelty following surgery in mice. In this study, sociability and preference for social novelty were assessed using the three-chamber test. Mice that underwent surgery (PB-POD1) exhibited normal sociability and no preference for social novelty on postoperative day 1. On postoperative day 7, operated mice (PB-POD7) showed normal sociability and preference for social novelty. Overall, these findings suggest normal social behavior in mice following surgery.

Depression was assessed using the tail-suspension test. Mice that underwent surgery (PB-POD1 and PB-POD7) showed depression-like behavior on postoperative days 1 and 7, as they had higher immobility times compared to their non-surgical counterparts (PA-POD1 and PA-POD7). Animal models of peripheral inflammation reported depression-like behavior, and attributed the phenomenon to a high turnover rate of brain serotonin as a result of an exaggerated elevation in inflammatory mediators in the brain [66,67]. On the contrary, depression was not shown to occur after surgery in humans [8,68,69], but it is considered to be a risk factor for POCD [47] and postoperative delirium [70,71].

As mentioned, the pathophysiology underlying POCD is not yet fully understood, and several etiological factors have been proposed. Hypocarbia due to hyperventilation during anesthesia was shown to prolong cognitive dysfunction after surgery [72], but other studies failed to establish the link [73,74]. Prolonged hypotension in the perioperative period was thought to result in cerebral hypoperfusion, and subsequently cognitive decline. However, the effect was not shown to be important [8,75,76,77]. Cognitive decline is well-documented following cardiac surgery [78,79,80], as it could be a source of microemboli (clots, fat, or air bubbles) that causes brain infarcts. However, POCD is often found following non-cardiac surgeries, and the presence of microemboli is not clearly linked to cognitive dysfunction [17,81,82]. Sleep disturbances following surgery can affect cognitive function, but evidence for such an effect is scarce [83]. Neurodegeneration can occur as a result of the use of volatile anesthetics, as shown by animal studies [84,85,86], but several other studies failed to find a link [17,22,23,38,87]. Other etiologies, including anticholinergic activity of medications routinely used in the perioperative period, and low intraoperative cerebral oxygenation, were also proposed, but the results were inconclusive [15].

Consistent evidence exists only for the role of postoperative peripheral inflammation in causing exaggerated neuroinflammatory responses in the brain, that manifest as cognitive decline. Severe systemic inflammation, as a result of trauma or infection, is known to affect the central nervous system (CNS) [88,89,90], and surgery was shown to result in the systemic release of inflammatory mediators, such as cytokines, reactive oxygen species, and endothelins [14,91,92,93,94,95]. These changes lead to the activation of microglia in the CNS, which results in the release of cytokines and other inflammatory mediators that have been linked to cognitive dysfunction [19,20,21,23,96,97]. Moreover, the severity of the surgical intervention is correlated to the risk of cognitive dysfunction postoperatively, which supports the inflammatory hypothesis, as more severe procedures are related to higher magnitudes of immune activation [14,17,23,24,98,99,100].

Interestingly, these neuroinflammatory responses also lead to neurodegenerative changes, such as tau phosphorylation, cytokine-induced glutamatergic excitotoxicity, synaptic impairment, and most importantly, beta-amyloid accumulation, which are also characteristic of Alzheimer’s disease [22,25,26,101,102]. It comes as no surprise that POCD have been linked to an increased risk of developing dementia [12] and, therefore, both entities can be viewed as manifestations of the same process [15]. It should be noted that the absence of neurofibrillary tangles in POCD distinguishes it from Alzheimer’s disease. Therefore, cognitive deficits in POCD are thought to be specifically related to beta-amyloid accumulation [25]. Beta-amyloid proteins are known to induce neuronal cell degeneration and apoptosis, especially in the hippocampus, resulting in memory impairment [102]. Additionally, amyloid precursor protein (APP), whose proteolysis leads to the generation of beta-amyloid proteins and acts as an acute reactive protein during stress [103,104], was also found to be increased following surgery in mice [25].

Management of POCD is centered around preventive measures. It involves identifying those who are at risk, and optimizing perioperative physical and mental health. Several measures have been shown to reduce the incidence of POCD [18,27,28,29,105,106,107]. These include improving sleep hygiene in the perioperative period, having shorter periods of fasting before surgery, increasing the frequency of social contact with family and friends after surgery, optimization of nutritional status and hydration, selecting the least invasive procedures possible, and adequate pain control.

However, no effective pharmacological treatment exists. Research in this area focused mostly on anti-inflammatory medications, since neuroinflammation contributes to POCD significantly. For example, the administration of small doses of ketamine, which has an anti-inflammatory effect, was shown to reduce the incidence of POCD [108]. Minocycline [23,109], a derivative of tetracycline, and berberine [49], an isoquinoline alkaloid, were also shown to reduce neuroinflammation in mice. In Alzheimer’s disease, the use of non-steroidal anti-inflammatory medications has been shown to attenuate disease progression in the early stages [110].

In the present study, we hypothesized that memantine, an NMDAR antagonist, that is shown to be effective in treating Alzheimer’s disease [30,31,32,33,34], might have a protective effect against POCD. The release of pro-inflammatory cytokines in the CNS leads to an increase in the levels of glutamate, which increases NMDAR activity. That, in turn, increases Ca^2+^ inflow through ion channels, which results in pathological over-excitation, and ultimately neuronal death. Furthermore, hyper-activation of NMDARs has also been associated with tau toxicity [111]. As mentioned previously, both of these processes, glutamatergic excitotoxicity and tau phosphorylation, have been shown to occur in Alzheimer’s disease and in POCD [25]. Memantine inhibits NMDARs by blocking the Ca^2+^ ion channel, and thus it targets both of the pathological processes that are known to be involved in POCD. Previous research also showed that memantine reduces neuroinflamamtion in animal models and improves cognitive function [112,113,114]. In our study, memantine improved the preference for social novelty, and had an anti-depressant-like effect, with no effect on memory or anxiety-like behavior on postoperative day 1. On day 7 postoperatively, memantine had a positive effect on memory and depression. In addition, memantine did not impact the body weight of treated mice. In humans, a randomized clinical trial conducted by Ghaffary and colleagues [115] showed that the pre-operative administration of memantine protected patients from POCD following cardiac surgeries, and improved cognitive function after 3 months postoperatively. Collectively, these findings suggest that memantine has the potential to prevent POCD in humans.

## 5. Conclusions

The findings of this study suggest a protective effect of memantine on memory, preference for social novelty, and depression-like behavior in mice following surgery. However, the study offers a coarse behavioral assay in mice, and further research is recommended to address the mechanisms by which memantine impacts POCD, and the feasibility of using it in humans.

## Figures and Tables

**Table 1:** Body weight (g). Data shown as mean ± standard error of the mean (SEM), *p* values given for t-tests.

Day ^1^	PA (n = 20)	PB (n = 20)	M (n = 20)
1	29.5 ± 0.9	30.1 ± 0.8	29.9 ± 0.9
7	30 ± 0.4	31 ± 0.9	30 ± 0.9
14	28.3 ± 0.8	27.9 ± 0.9	29 ± 1
21	31.2 ± 0.5	29.9 ± 1.1	31.3 ± 0.8
28	27.9 ± 0.9	29 ± 1.1	28.9 ± 0.9
29 (POD1)	30.1 ± 0.9	27 ± 1.1 *	26.2 ± 0.9 **
31 (POD3)	31 ± 1	26 ± 0.9 **	26.6 ± 0.8 **
35 (POD7)	29.5 ± 1.1	31 ± 1	30.1 ± 1.1

^1^ Days 1–28: treatment period. POD: postoperative day * *p* < 0.05 as compared to PA. ** *p* < 0.01 as compared to PA.

**Table 2:** The Morris water maze. Data shown as mean ± SEM, *p* values given for t-tests.

Group ^1^	Latency (s)	Time in Disc Zone (%)	Distance Swum (dm)	Speed of Swimming (m/s)
PA-POD1	42.2 ± 6.3	30.4 ± 1.2	8.6 ± 2	0.22 ± 0.02
PB-POD1	61.2 ± 7.7 **	21.6 ± 1.4 **	14.7 ± 2.6 *	0.23 ± 0.01
M-POD1	54.4 ± 4.4 **	22.7 ± 4 *	8.3 ± 1	0.25 ± 0.02
PA-POD7	45 ± 9	35.4 ± 2.5	6.2 ± 1	0.27 ± 0.02
PB-POD7	67.7 ± 4.6 ^†^	24.6 ± 3.2 ^††^	10.5 ± 1.1 ^††^	0.26 ± 0.02
M-POD7	47.3 ± 4.7	35.3 ± 4	5.2 ± 0.6	0.25 ± 0.02

^1^ Each group (n = 10). * *p* < 0.05 as compared to PA-POD1. ** *p* < 0.01 as compared to PA-POD1. ^†^ *p* < 0.05 as compared to PA-POD7. ^††^ *p* < 0.01 as compared to PA-POD7

**Table 3:** The open field test. Data shown as mean ± SEM, *p* values given for t-tests.

Group ^1^	Number of Lines Crossed	Frequency of Central Area Entry	Time Spent at Central Area (s)
PA-POD1	92.8 ± 2.9	6.2 ± 0.6	19.5 ± 4.3
PB-POD1	105.6 ± 3.4	6.8 ± 1	10.5 ± 1.9 **
M-POD1	99.9 ± 10.2	6.1 ± 1.2	6.9 ± 1.4 *
PA-POD7	127.6 ± 4.6	7.4 ± 0.9	12.2 ± 1.3
PB-POD7	119.1 ± 6.5	8.6 ± 1.2	14.3 ± 1.8
M-POD7	122 ± 11.2	7.6 ± 1.3	13.7 ± 2.6

^1^ Each group (n = 10). * *p* < 0.05 as compared to PA-POD1. ** *p* < 0.01 as compared to PA-POD1

**Table 4:** The elevated plus-maze. Data shown as mean ± SEM, *p* values given for t-tests.

Group ^1^	Time Spent in Open Arms (s)	Open Arms Entries (%)	Total Number of Arm Entries
PA-POD1	121.3 ± 6.4	45.7 ± 2.4	20.7 ± 0.8
PB-POD1	67.1 ± 19.5 *	32.1 ± 3.1 **	22.6 ± 2.4
M-POD1	64.2 ± 10.5 **	29.4 ± 2.3 **	21.8 ± 2
PA-POD7	133.2 ± 6.9	47.7 ± 4.6	22.8 ± 2
PB-POD7	101.5 ± 7 ^†^	31.8 ± 2.7 ^†^	22.9 ± 1.7
M-POD7	93.1 ± 6.5 ^†^	29.3 ± 2.4 ^†^	25.2 ± 1.2

^1^ Each group (n = 10). * *p* < 0.05 as compared to PA-POD1. ** *p* < 0.01 as compared to PA-POD1. ^†^ *p* < 0.01 as compared to PA-POD7

**Table 5:** The tail-suspension test. Data shown as mean ± SEM, *p* values given for t-tests.

Group ^1^	Immobility Time (s)
PA-POD1	119.1 ± 16.4
PB-POD1	180.3 ± 3.5 **
M-POD1	131 ± 9.2
PA-POD7	34.8 ± 4.3
PB-POD7	58.3 ± 6.2 ^†^
M-POD7	37.6 ± 5.9

^1^ Each group (n = 10). ** *p* < 0.05 as compared to PA-POD^1^. ^†^ *p* < 0.05 as compared to PA-POD7.

**Table 6:** Time spent in chambers in the three-chamber test. Data shown as mean ± SEM, *p* values given for t-tests.

Group ^1^	Session 1		Session 2	
	Time with Mouse (s)	Time without Mouse (s)	Time with Old Mouse (s)	Time with New Mouse (s)
PA-POD1	299.7 ± 20.8 **	190 ± 21.4	190.2 ± 14	290 ± 18.2 ^††^
PB-POD1	286.1 ± 27.5 *	209 ± 31.3	259.6 ± 22.2	272.5 ± 22
M-POD1	309.7 ± 28 **	193 ± 24.6	170 ± 18.4	345.2 ± 26.7 ^††^
PA-POD7	314.4 ± 20 **	176 ± 22.2	233.4 ± 28.8	280 ± 22 ^††^
PB-POD7	325 ± 22.1 **	178.2 ± 19.9	232 ± 22	288.4 ± 26.6 ^††^
M-POD7	322.2 ± 26.2 **	178 ± 19.2	225.3 ± 26.1	277.2 ± 25 ^††^

^1^ Each group (n = 10). * *p* < 0.05 as compared to time without mouse. ** *p* < 0.01 as compared to time without mouse. ^††^ *p* < 0.01 as compared to time with old mouse

**Table 7:** Frequency of entry to chambers in the three-chamber test. Data shown as mean ± SEM.

Group ^1^	Session 1		Session 2	
	Chamber with Mouse	Chamber without Mouse	Chamber with Old Mouse	Chamber with New Mouse
PA-POD1	7.3 ± 0.6	7.9 ± 1.4	7.7 ± 0.5	8.7 ± 0.4
PB-POD1	6.4 ± 0.8	5.4 ± 0.7	9.9 ± 1.5	10.4 ± 1.8
M-POD1	8.3 ± 0.6	7.1 ± 1	8.4 ± 0.7	7.9 ± 0.4
PA-POD7	7.4 ± 0.5	5.8 ± 1	6.9 ± 0.4	7 ± 0.5
PB-POD7	7.2 ± 0.6	6.1 ± 0.4	6.5 ± 0.4	7.6 ± 0.4
M-POD7	12 ± 1.2	14.6 ± 1.5	13.4 ± 0.9	14.3 ± 1.4

^1^ Each group (n = 10).
